# On the effect specificity of accessory gland products transferred by the love-dart of land snails

**DOI:** 10.1186/s12862-016-0672-6

**Published:** 2016-05-13

**Authors:** Monica Lodi, Joris M. Koene

**Affiliations:** Section of Animal Ecology, Department of Ecological Science, VU University Amsterdam, De Boelelaan 1085, 1081HV Amsterdam, The Netherlands; Naturalis Biodiversity Center, Darwinweg 2, 2333 CR Leiden, The Netherlands

**Keywords:** Accessory gland protein, Allohormone, Contraction, Mollusc, Sexual selection, Simultaneous hermaphrodites

## Abstract

**Background:**

Sexual selection favours the evolution of male bioactive substances transferred during mating to enhance male reproductive success by affecting female physiology. These effects are mainly well documented for separate-sexed species. In simultaneous hermaphrodites, one of the most peculiar examples of transfer of such substances is via stabbing a so-called love-dart in land snails. This calcareous stylet delivers mucous products produced by accessory glands into the mate’s haemolymph. In *Cornu aspersum*, this mucus temporarily causes two changes in the recipient. First, the spermatophore uptake into the spermatophore-receiving organ, called diverticulum, is probably favoured by contractions of this organ. Second, the amount of stored sperm increases by contractions of the copulatory canal, which close off the tract leading to the sperm digesting organ. However, it has yet to be determined whether these effects are similar across species, which would imply a common strategy of the dart in increasing male reproductive success.

**Results:**

We performed a cross-reactivity test to compare the *in vitro* response of the diverticulum and copulatory canal of *C. aspersum* (Helicidae) to its own and other species’ mucus (seven helicids and one bradybaenid). We found that the contractions in the diverticulum were only induced by dart mucus of certain species, while the copulatory canal responded equally to all but one species’ mucus tested. In addition, we report a newly-discovered effect causing the shortening of the diverticulum, which is also only caused by dart mucus of certain species. The advantage seems to be a distance reduction to the sperm storage organ.

**Conclusions:**

All these findings are the first to shed light on the evolution of the different functions of accessory gland products in dart-bearing species. These functions may be achieved via common physiological changes caused by the substances contained in the dart mucus, since the responses evoked were similar across species’ mucus. Moreover, while these substances can act similarly in separate-sexed species as in simultaneous hermaphrodites, differences may occur in their evolution between the two sexual systems.

**Electronic supplementary material:**

The online version of this article (doi:10.1186/s12862-016-0672-6) contains supplementary material, which is available to authorized users.

## Background

The transfer of male bioactive substances during mating is ubiquitous [1, 2]. So far these substances are especially well characterised in the genus *Drosophila*, being highly divergent and species-specific [3–9]. The evolution of these substances is favoured by sexual selection since they enhance male reproductive success by affecting the behaviour or physiology of the female. Changes in the female body, such as inducing egg laying [10] and decreasing female willingness to remate [11], are common examples of such male manipulation. However, these effects might contrast with the interests of females over reproduction, leading to sexual conflict [2, 12].

In internal fertilizers, male manipulation is achieved in two ways. The bioactive substances are either transferred along with the sperm in the seminal fluid or separately [13]. A way to employ the latter strategy is to inject the substances through the partner’s skin [14]. This occurs both in separate-sexed and hermaphroditic species (such as scorpions, salamanders, earthworms, sea slugs; [13]). One of the most prominent examples is the love-dart of simultaneously hermaphroditic land snails (gastropods), which has received growing attention recently (e.g. [15–17]).

**Fig. 1:**
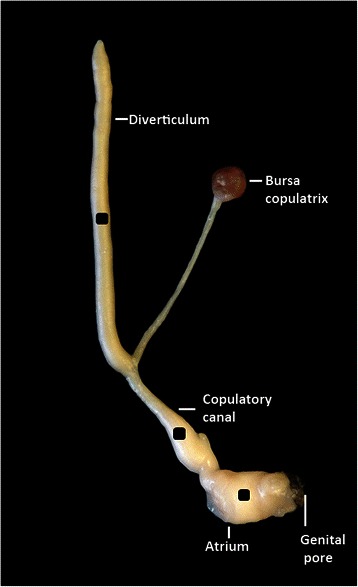
The part of the reproductive system of *C. aspersum* that was used in this study*.* This includes the genital pore where the partner’s spermatophore enters; atrium; copulatory canal; the bursa copulatrix which is responsible for sperm digestion; the diverticulum which receives the spermatophore. This is the preparation used for each experimental trial. The three black squares (±2 mm^2^) of electrical tape glued onto diverticulum, copulatory canal and atrium were used as markers. The position of these markers was recorded with a webcam, then tracked with DLTdv5 marker tracking software to measure the response of the preparation to each species mucous extracts added to the saline bath (see Additional file 1: Movie 1). Not illustrated: the small dish containing 2 ml saline solution where the preparation was placed and the pins on both sides along the length of the diverticulum, in the Sylgard base of the small dish, to make the measurements comparable

Simultaneously hermaphroditic snails are male and female at the same time and the love-dart is a stylet-like structure that is mainly made of a crystalline form of calcium carbonate and hosted in a muscular dart sac [18]. The morphology of darts depends on the species and so does the number of darts possessed (reviewed by [19]). During courtship, the dart is pushed out of the sac (referred to as dart shooting behaviour), exits via the genital pore and pierces the right flank of the partner’s body wall. When the dart is expelled, it is coated with mucus produced by accessory glands that are located adjacent to the dart sac [18]. Once this mucus is introduced into the haemolymph of the partner, it is distributed throughout the circulatory system [20]. For *Cornu aspersum* (Helicidae), which is the only species investigated *in vitro* in this respect, the mucus has been reported to cause temporary changes of two female reproductive organs of the partner [21]. The first occurs in the diverticulum, a blind-ended duct receiving the spermatophore, which is connected to the tract leading to the bursa copulatrix, the sperm digesting organ (Fig. 1). Depending on the species, the diverticulum can be present or not [22]. When absent, the spermatophore of the mate is received either in the bursa copulatrix tract or in the oviduct [22]. The mucus has been shown to increase the number of contractions in the diverticulum, probably allowing an easier uptake of the spermatophore [21]. The second change occurs in the copulatory canal (called pedunculus of the bursa copulatrix by [23]), which bifurcates into the diverticulum and the bursa copulatrix tract connecting them to the atrium, placed behind the genital pore (Fig. 1). Under the influence of mucus, contractions of the copulatory canal make the entrance to the bursa copulatrix tract less accessible, permitting more sperm to avoid digestion and reach the sperm storage organ [21]. As a result, the successful dart shooter more than doubles its paternity in the partner’s eggs [24].

The above changes represent the better known ways of affecting the partner physiology via the action of the mucus. Other examples are found in *Euhadra quaesita* (Bradybaenidae), whose dart mucus suppresses the partner’s willingness to remate and induces egg laying in small individuals [25]. All these findings suggest a common function of the dart in enhancing male reproductive success across families. Whether this function is achieved via common physiological changes in different species remains unknown.

To gain insight into the evolution of male manipulative function across species, we investigated whether the effects induced by mucus carried on the love-dart in land snails are conserved or species-specific. We did so by assessing the response to dart mucus using a cross-reactivity test. This test is important to assess the diversity in effects of mucous products in related species, which would be missed when mucus was tested on the species itself. Thus, the mucus of the species tested might not cause the same reaction when applied to their own reproductive organs. This may be due to counter-adaption to the bioactive substance taking place, making the female organs less reactive, which usually occurs in response to sexual conflict [26]. It should be noted that with our approach we cannot distinguish whether the observed effects are caused by similar substances; mucous products can be different between species but still maintain the same manipulative function [9, 27, 28].

Hence, for this study, we used seven species of the Helicidae and one of the Bradybaenidae family to test whether the response of the female reproductive tract of *C. aspersum* (Helicidae) to dart mucus is the same. This was done by comparing the *in vitro* reaction of *C. aspersum*’s diverticulum and copulatory canal to mucous accessory gland products of these different species. We also present the new technique developed to quantify the physiological response and its intensity. In addition, we report a newly-discovered effect of the mucus on the diverticulum.

## Methods

### Study species

Snails of the species *Cepaea nemoralis*, *Cepaea hortensis* and *Arianta arbustorum* were collected between June and September 2013 in Almere, the Netherlands. *Cornu aspersum*, *Helix pomatia*, *Theba pisana* and *Eobania vermiculata* were obtained from the snail farm EuroHelix Chierasco, Italy in September 2013. The species *Helix lucorum* and *Fruticicola fruticum* were acquired from Thessaloniki, Greece in October 2013. These species belong to the Helicidae family besides the bradybaenid *F. fruticum*. All species were kept at 20 °C with a reversed photoperiod L:16 h D:8 h at 60 % humidity. Adult snails were kept individually in plastic boxes lined at the bottom with moist paper (*Helix pomatia* and *Helix lucorum*: 17.5 cm x 11 cm x 13 cm; the smaller species: 11.5 cm x 11.5 cm x 5 cm), and isolated for at least two weeks to prevent them from mating and consequently empty their mucous glands. The snails were cleaned, fed with lettuce and snail feed as a source of calcium twice a week (the Chase mix; R. Chase, personal communication: 50 % chicken feed for growing chickens and 50 % grain mix of calcium carbonate 18 %, soya protein 10 %, wheat flour 20 %, wheat bran 10 %, corn flour 16 %, barley flour 16 %, ground sunflower seeds 6 %, calcium phosphate 2 %, ground vitamin mix 1 %, methyl paraben to retard mould 1 %).

### Cross-reactivity test

On each experimental day, one *C. aspersum* snail was anesthetized with 50 mM MgCl_2_ and the genital atrium, diverticulum, bursa copulatrix and its tract were dissected out. Besides the atrium, these organs were also the targets of stimulation by *C. aspersum* mucus [21] (the atrium, leading up to the genital pore, was included in case it was activated by mucus of other species). These organs, hereafter jointly referred to as the "preparation”, were kept in a small dish containing 2 ml of saline solution with pH 7.8 (control saline [29]), equivalent to the amount of haemolymph of *C. aspersum* [30]. In order to have comparable measurements of the diverticulum’s movements, several pins were placed on both its sides in the Sylgard base of the small dish (see also [21]). Subsequently, three squares of black electrical tape of approximately 2 mm^2^ were glued onto the diverticulum, copulatory canal and atrium with tissue adhesive (®TA5). These black squares will be referred to as “markers” (Fig. 1).

Each mucous gland extract was obtained by dissecting the mucous glands associated with the dart sac out of one anesthetized snail and crushing them with a plastic pestle in 0.5 ml saline solution. At the beginning of each experimental day, before testing the different types of mucous extracts, the preparation had a 30 min. adjustment period in the saline. Then, portions of gland extracts were tested by addition to the saline bath of the preparation. Every portion contains 2.2 mg of mucus, which represents a biological relevant dose since it is equivalent to the amount of mucus carried by the dart of *C. aspersum* (this amount was calculated by subtracting the dart’s wet weight before and after dart shooting [21]). The mucous extract of *C. aspersum* was used as positive control once at the beginning and once at the end of each experimental day to check whether there was variation in response over time for each organ. The mucous extracts of five other species were chosen randomly as well as the order in which they were tested between the two controls. This means that not all the species were tested for each preparation and this created different sample sizes between species. To prevent pseudoreplication each extract was used only once.

For each trial, i.e. each mucus tested, the control activity of the preparation was recorded for 10 min. with a webcam (Logitech® HD Pro Webcam c920). Subsequently, a mucous extract was added and allowed to take effect for 5 min. before recording the response activity for another 10 min. [21]. Between trials the preparation was washed three times with saline, and allowed to rest for 5 min. in new saline solution. In total, each preparation was used for 3.5 h, comparable to the amount of time employed by Koene and Chase [21] in their experiments with the preparations.

**Fig. 2:**
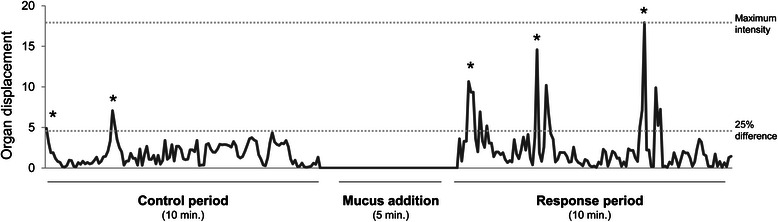
Example of a video track. The x-axis shows the duration of each trial where a control period is recorded as well as a response period after 5 min. pause in which the mucous extract was added. The y-axis indicates a measure of organ displacements (it can be approximately 1.5 cm for the diverticulum and approximately 1 cm for the copulatory canal). This measure is based on the coordinate output of the marker tracking software, expressed in millions, and shown on the y-axis as values divided by 10^6^. The displacements are counted as contractions and marked with asterisks. In this case the mucus induced one more contraction compared to the control. The maximum intensity reached by the contractions is shown by a dotted line. To count only relevant displacements, a threshold of 25 % difference was fixed between the minimum and maximum point for each trial (dotted line)

The videos recorded were analysed with DLTdv5 software [31], which was set to auto-track the position of the markers for each frame. The resulting output coordinates were used to obtain graphs of the displacement of the markers every 5 sec., where the difference between time points was calculated with the Euclidian distance between two points. The measurements obtained with these graphs are: number of contractions induced by mucus, calculated as difference between numbers of contractions counted in the response period and control period; intensity of contractions to assess the potency of the extracts, measured as the maximum displacement reached in each trial (maximum displacement of the diverticulum can be approximately 1.5 cm, equal to the distance between the pins placed along the sides of the diverticulum, and approximately 1 cm for the copulatory canal); percentages of times each organ reacted to the different types of mucus based on the instances that the number of contractions in the response period were higher than the ones in the control period. Some additional criteria were applied in order to count relevant contractions (see also [21]): first, a threshold of 25 % difference was fixed between the minimum and maximum point for each trial; second, contractions that lasted more than 3 min. were not counted; third, peaks of contractions had to be separated at least 15 sec. Figure 2 shows an example of a video track used in our analyses and Movie 1 (see Additional file 1) an example of the activity of the preparations recorded.

### Shortening effect on diverticulum

Although it was not the main focus of the current study, while performing the experiment an effect never described before caused by the mucus was observed: within seconds from the addition of certain species’ mucous gland extract, the diverticulum became shorter (see Additional file 2: Movie 2). This effect remained consistent throughout the response period. Hence, two pictures of the preparation were taken with the webcam, once after the control period (when the mucous extract was not added yet) and once after the response period (15 min. later). These pictures were used to assess the length of the diverticulum with ImageJ by measuring its length from the tip to the branching of the bursa copulatrix tract. Three length measurements of the diverticulum were made on each side of the organ, and the average of these measurements was used. Percentages of length reduction caused by each species’ mucus were also calculated.

### Phylogeny

To address the phylogenetic relationship between the species used in this study, the maximum likelihood (ML) method was applied by using partial 28 s nuclear gene sequences (sequences by [32]). *Discus rotundatus* was chosen as outgroup and *Euhadra sandai* was added as a second species (besides *Fruticicola fruticum*) from the Bradybaenidae family. The ML tree was built with MEGA5 following the protocol of Hall [33]. The sequences were aligned with MUSCLE, resulting in 726 reliably aligned nucleotide positions. The Tamura-3-parameter with a gamma distribution was the best substitution model obtained by ranking the models by the lowest Bayesian Information Criterion (BIC). Partial deletion of gaps/missing data was applied and to estimate the reliability of the tree, 1000 bootstraps were performed. Note that a full reconstruction of a phylogeny of land snails goes beyond our purpose here; our intention is to use the phylogenetic information to test for phylogenetic signal in our data. This would indicate whether mucus of closely-related species resemble each other in their response.

### Statistical analyses

Data from the cross-reactivity test were analyzed as follows. Since the atrium showed very low reaction, also in response to other species’ mucus, it was excluded from further analyses as a non-meaningful response. Data from experimental days were left out from the analysis when either the diverticulum or copulatory canal did not respond to any species’ mucus (this happened twice for each organ; these preparations responded normally to the control). To account for dependency in our data, we included potential order effects by testing, within a preparation, whether the species’ mucus tested before affected the response to a specific species mucus. We did so using a Generalized Linear Model (GLM) with Poisson distribution and Log link function for the number of contractions, and a normal distribution and Log link function for the intensity of contractions. In these analyses we included Species and Previous species tested as factors and compared the models with or without the second factor based on the Akaike criterion. To check whether the physiological preparations differed in their response (number of contractions and their intensity for both diverticulum and copulatory canal) across experimental days, we performed a Kruskal-Wallis test to compare days. To test whether for each organ there was variation in number and intensity of contractions between the two time points in which the positive control was tested, we performed a Wilcoxon test for paired observations. For the other mucus types, we tested whether there was a difference in response depending on testing order using a Kruskal-Wallis test.

To test whether the number and intensity of contractions in response to different mucus types diverged from the response to *C. aspersum* mucus, multiple comparisons were performed with the Steel method with control [34] because these data were not normally distributed. This test compares each species to the control species and is thus the non-parametric version of Dunnett’s test with control. The percentage of times each organ reacted to different types of mucus was compared to the average percentage response of the two positive controls with a Chi-square test.

Data on the shortening effect were repeated measures since the diverticulum length was measured at two time points. Since not all groups were normally distributed and in order to include them all in the same statistical test, we log-transformed these data. Then, we performed a mixed ANOVA to test the effect of time on the length of the diverticulum. This test is regularly used to compare means between two or more independent variables of which one is a repeated measure [35]. Our two independent variables were Time and Species of which Time is the repeated measure. In case of significant interaction, we calculated the simple effect with Fisher’s LSD adjustment to reveal for which species the factor Time had a significant effect. This test makes pairwise comparisons between the variable Time for each species. Finally, to test for dependency in our data, we performed a one-way ANOVA on diverticulum length at time 0 to assess if this organ regained its original length after each trial.

With the phylogenetic reconstruction, we tested for a phylogenetic signal in the response to mucus (number and intensity of contractions for both diverticulum and copulatory canal) and in the shortening effect (actual shortening in mm) by using Blomberg’s *K* with the *phylosig* function in the R package *phytools* [36]. *K* indicates the degree at which a trait shows phylogenetic signal predicted under Brownian evolution (*K* = 0 means that there is no phylogenetic signal, *K* < 1 means that closely-related species weakly resemble each other, and *K* > 1 indicates that closely-related species strongly resemble each other) [37]. To obtain p-values of *K* we used 1000 randomization.

## Results

### Cross-reactivity test

The mean number ± SD of contractions induced by the mucus of the two *C. aspersum* positive controls, per 10 min. recording, is 1.98 ± 2.23 for the diverticulum (*N* = 58), 1.28 ± 1.78 for the copulatory canal (*N* = 58) and 0.57 ± 1.25 for the genital atrium (*N* = 58).

The mucus type tested, i.e. the factor Species, had a significant effect on the response of the diverticulum (GLM: number of contractions, d.f. = 8, *p* = 0.005; intensity, d.f. = 8, *p* < 0.001) but not of the copulatory canal (GLM: number of contractions, d.f. = 8, *p* = 0.134; intensity, d.f. = 8, *p* = 0.094), indicating that the variance in the response of the latter was similar between species. Moreover, these analyses showed that our data points were independent from each other, since the response to a species’ mucus was not influenced by the mucus type tested before (factor Previous species tested). This was neither the case for the diverticulum (GLM: number of contractions, d.f. = 8, *p* = 0.132; intensity, d.f. = 8, *p* = 0.144) nor for the copulatory canal (GLM: number of contractions, d.f. = 8, *p* = 0.233; intensity, d.f. = 8, *p* = 0.561). For these diverticulum tests, the Akaike score was better for the model without the second factor for the contractions (AICc: 221.4 and 236.8, respectively) but not for the intensity (AICc: 5176.7 and 5169.9, respectively). For the copulatory canal, the Akaike scores were lower for both models without the second factor (AICc: contractions, 271.0 and 290.9; intensity, 5678.5 and 5687.0, respectively).

**Fig. 3:**
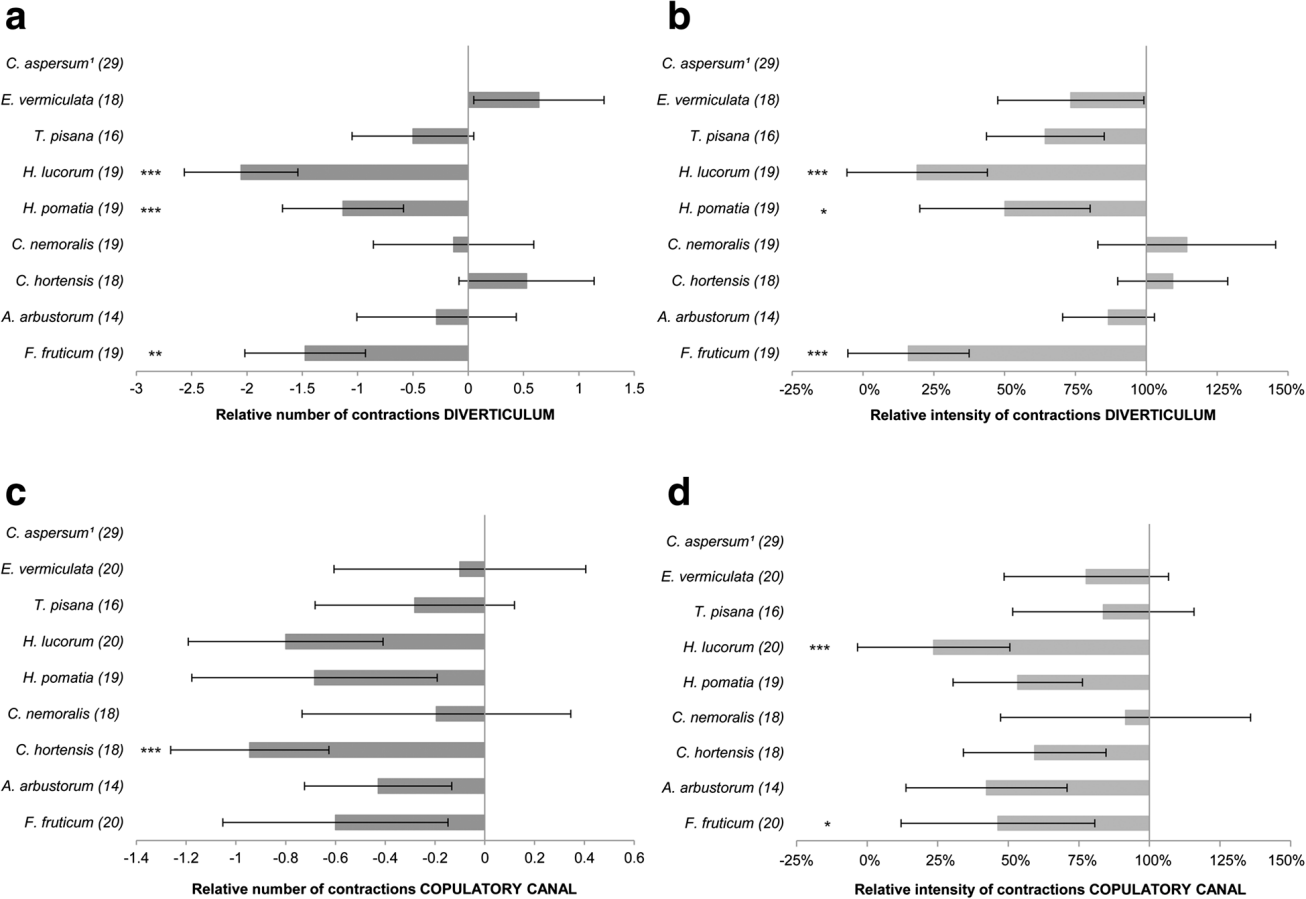
Graphs illustrating the response of *C. aspersum* to each species’ mucus. **a** Relative number of contractions of the diverticulum. **b** Relative intensity of contractions of the diverticulum. **c** Relative number of contractions of the copulatory canal. **d** Relative intensity of contractions of the copulatory canal. Mean ± SE is given for each graph. The graphs are obtained by subtracting the mean response of the control species from the response of each species mucous extract for every experimental day. Note that the intensity is shown here as percentages of displacement compared to the control species (e.g. lower percentages indicate a relatively lower response); the control species has 100 % response and 0 % response is based on coordinates of preparations of the species showing zero response. The symbol ^1^ indicates that *C. aspersum* is the baseline hence its bar mean is zero. Numbers in parentheses indicate the sample size and the asterisks show the significance according to the Steel method (*p < 0.05, **p < 0.01, ***p < 0.001). For ease of comparison, species are listed according to their order of appearance on the phylogenetic tree

Overall, we did not find an effect of the experimental day (*N* = 7 per day, Days = 29) on either the response of the diverticulum (Kruskal-Wallis: number of contractions, H = 37.226, *p* = 0.114; intensity, H = 35.863, *p* = 0.146) or the copulatory canal (Kruskal-Wallis: number of contractions, H = 32.546, *p* = 0.253; intensity, H = 31.719 *p* = 0.286). The two time points in which the positive control was tested on the same experimental day differed only in terms of number of contractions of the diverticulum (control 1 = 1.48 ± 2.32, control 2 = 2.48 ± 2.04; Wilcoxon: Z = −1.992, *p* = 0.046), with the second control being higher, while its intensity showed a trend in the same direction (control 1 = 2.4x10^6^ ± 4.6x10^6^, control 2 = 4.0x10^6^ ± 5.2x10^6^; Wilcoxon: Z = −1.894, *p* = 0.058). However, no such pattern could be found in the testing order of the other mucus types (Kruskal-Wallis: H = 2.086, *p* = 0.72). To overcome the variation of the diverticulum response between the two time points, the response of the preparation to each mucus type (sample sizes are indicated in Fig. 3) was expressed as relative response: the response minus the mean response of the two controls on that experimental day. The relative number of contractions of the diverticulum as well as the relative intensity differed in the three species *H. lucorum*, *H. pomatia* and *F. fruticum* (Steel method: number of contractions *H. lucorum*, Z = −4.83, *N* = 19, p < 0.001; *H. pomatia*, Z = −4.36, *N* = 19, *p* = 0.0001; *F. fruticum*, Z = −3.34, *N* = 19, *p* = 0.0064; intensity: *H. lucorum*, Z = −4.08, N = 19, *p* = 0.0004; *H. pomatia*, Z = −2.90, *N* = 19, *p* = 0.0267; *F. fruticum*, Z = −4.08, *N* = 19, *p* = 0.0004), with all of them being lower than the positive control (Fig. 3a, b). Except for *C. hortensis* (Steel method: Z = −4.21, *N* = 18, *p* = 0.0002), the relative number of contractions of the copulatory canal induced by mucus types did not differ from *C. aspersum* mucus (Fig. 3c). For the relative intensity of the contractions, only *H. lucorum* and *F. fruticum* had lower intensity than *C. aspersum* (Steel method: Z = −4.50, *N* = 20, *p* < 0.001; Z = −2.72, *N* = 20, *p* = 0.0456, respectively) (Fig. 3d).

**Fig. 4:**
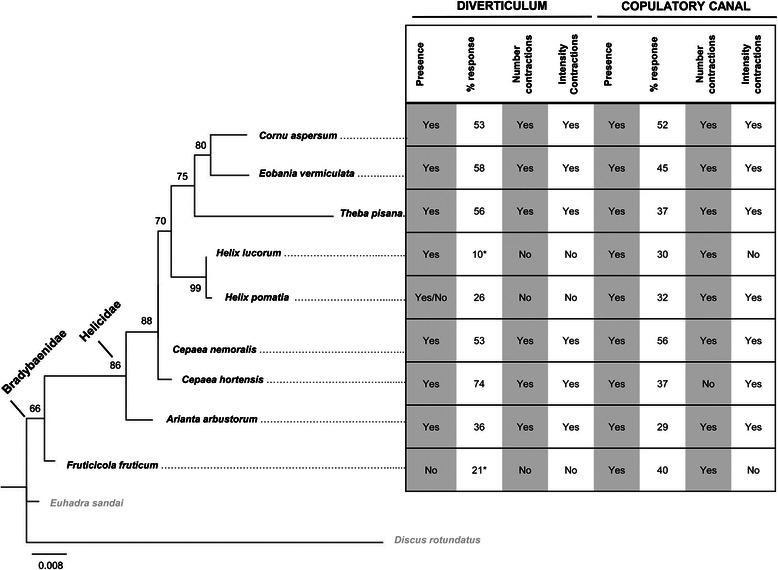
Summary of the results and phylogeny of the species used in this study. The ML phylogenetic tree is shown on the left side. The phylogeny is based on 726 nucleotide sites using Tamura-3-parameter model with a gamma distribution. Bootstrap values above 50 % support are given to the nodes (1000 replications). The length of branches refers to the estimated number of changes occurred between nodes (see scale bar). *Discus rotundatus* is the outgroup and *Euhadra sandai* was chosen to support the Bradybaenidae. On the right side, a summary table of the results for both diverticulum and copulatory canal is shown (each row corresponds to each species used in this study). The first column of each organ shows whether it is present (Yes), absent (No) or facultative (Yes/No). The second column shows the percentages of response to the mucous gland extracts for each species. For the focal species *Cornu aspersum,* the average response of the two time points the mucus was tested is given (once at the beginning and once at the end of each experimental day). The asterisks refer to the significant Chi-square test when that species was compared to the focal species. The last two columns refer to the results shown in Fig. 3 indicating whether the response was similar to the one of the focal species (Yes) or not (No)

The percentages of times the diverticulum and copulatory canal responded to each type of mucus are shown in Fig. 4. Only *H. lucorum* and *F. fruticum* diverticula reacted significantly fewer times compared to the control (*χ*^2^_1_ = 9.08, *p* = 0.003; *χ*^2^_1_ = 4.97, *p* = 0.026, respectively) and no differences between percentages of response of the copulatory canal were found.

### Shortening effect on diverticulum

**Fig. 5:**
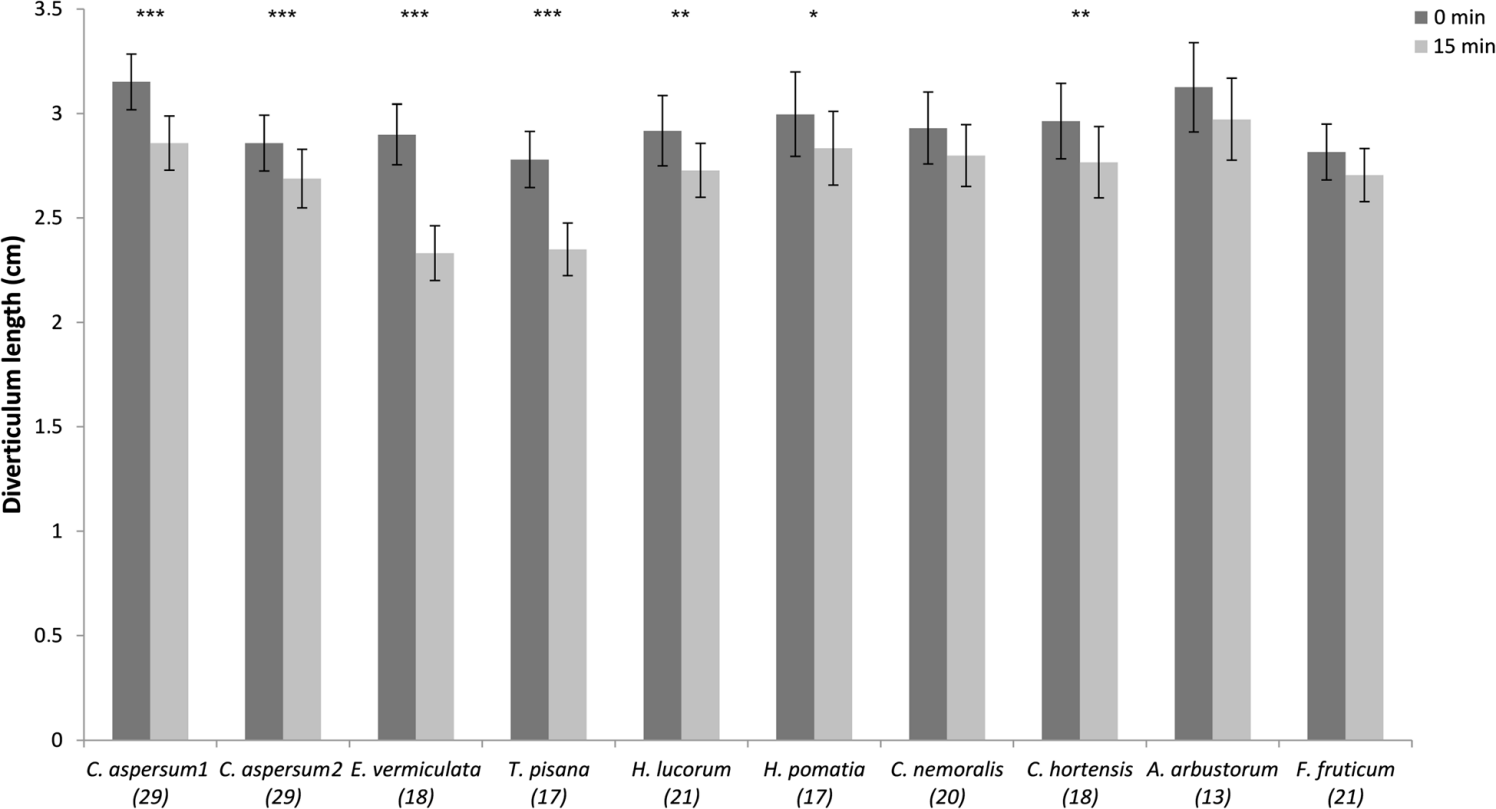
Shortening effect on *Cornu aspersum*’s diverticulum. Length (mean ± SE) of *Cornu aspersum*’s diverticulum before and 15 min. after the application of each species’ mucous gland extract (see Additional file 2: Movie 2 for an example). Numbers in parentheses indicate the sample size and the asterisks show the significance (*p < 0.05, **p < 0.01, ***p < 0.001). *C. aspersum* 1 and 2 refer to the two positive controls tested at the beginning and at the end of each experimental day

For the shortening response of the diverticulum induced by mucus (sample sizes are indicated in Fig. 5), there is an overall effect of Time and a significant Time x Species interaction (mixed ANOVA: F = 147.805, d.f. = 1, *p* < 0.001; F = 6.908, d.f. = 9, *p* < 0.001, respectively). This indicates that in general at 15 min. the diverticulum becomes shorter under the influence of mucus but the strength of this effect depends on which type of mucous extract was applied to the preparation. The greater shortening effect was specifically caused by the mucus of both *C. aspersum* positive controls (for each: *N* = 29, *p* < 0.001), *C. hortensis* (*N* = 18, *p* = 0.004), *H. pomatia* (*N* = 17, *p* = 0.035), *H. lucorum* (*N* = 21, *p* = 0.008), *T. pisana* (*N* = 17, *p* < 0.001) and *E. vermiculata* (*N* = 18, *p* < 0.001) (Fig. 5). The strongest length reduction was induced by the mucus of *E. vermiculata*, which makes the diverticulum of *C. aspersum* approximately 20 % shorter (Table 1). After performing each trial, the diverticulum regained its original length (ANOVA: F = 0.601, d.f. = 9, *p* = 0.795) permitting the measurements to have the same baseline and the data points to represent similar measurements.

**Table 1:** Length reduction of *Cornu aspersum*’s diverticulum. The table lists the species’ mucous gland extracts that caused significant shortening after addition to the saline bath. Length reduction is expressed as mean in mm and in percentages of the total length at time 0, i.e. before addition of the extracts

Species	Shortening (mm)	Shortening (%)
C. aspersum*	2.9-1.7	9.3-6.0
*C. hortensis*	2.0	6.6
*H. pomatia*	1.6	5.4
*H. lucorum*	1.9	6.5
*T. pisana*	4.3	15.5
*E. vermiculata*	5.7	19.6

The asterisk (*) refers to the two positive controls and their effect range

### Phylogeny

In order to assess the phylogenetic relationship between the species tested, we performed a phylogenetic reconstruction analysis that resulted in a tree (Fig. 4) that is in agreement with the latest Helicoidea phylogeny [38]. Features to point out include that the genus *Helix* is retrieved as a monophyletic group [39, 40] and the two Bradybaenidae species form a distinct group [40, 41] with relatively well supported nodes (bootstrap values: 99; 66, respectively).

We found no significant phylogenetic signal, neither for the response to mucus in both diverticulum (number of contractions: *K* = 0.25, *p* = 0.298; intensity: *K* = 0.38, *p* = 0.154) and copulatory canal (number of contractions: *K* = 0.10, *p* = 0.732; intensity: *K* = 0.10, *p* = 0.687), nor for the shortening effect (*K* = 0.67, *p* = 0.136).

## Discussion

By comparing the response of *Cornu aspersum* (Helicidae) to its own and other species’ mucous gland extracts, we found that the reactions of the copulatory canal and the diverticulum are not species-specific, since most species’ mucus induced a similar effect. An overview of the results and the phylogenetic relationship of the species used, eight helicids and one bradybaenid, is provided in Fig. 4.

The difference in response of the diverticulum among mucus types may be due to the morphological variation in this organ. In response to sexual conflict, this organ evolved as female counter-adaptation to the male manipulation of the love-dart [19, 32]. In this way the female increases the distance to the sperm storage organ and the diverticulum is further elongated in species that possess darts with a greater surface area (potentially holding more mucus) [32]. As a result, the presence and length of the diverticulum depends on the species, whereas the copulatory canal is always a standard component of the reproductive system. This implies that the significantly lower reaction to the mucus of *F. fruticum* (Bradybaenidae) might be due to this species lacking a diverticulum and receiving the spermatophore in the vaginal duct instead [42]. Within the Helicidae all species tested possess a diverticulum. Although in *C. hortensis*, *C. nemoralis* and *H. lucorum* it is very short, and in *H. pomatia* either it is short or absent, the length showing a geographical trend [43]. Interestingly, the response of the diverticulum was significantly lower only for the two closely-related *Helix* species*,* with short or no diverticula. Thus, the substance causing the contraction of the diverticulum might have evolved only in the Helicidae since it is the only family possessing such an organ. The two *Helix* species may either never have had such a substance, have gained and subsequently lost it, produce a lower concentration of it, or have evolved really different proteins to achieve the same effects in their own species. Alternatively, the substance targeting this organ evolved in ancestors of the Helicidae but is not shared by the Bradybaenidae. Clearly, an extended study including more species of both families would clarify this point.

For the copulatory canal, our video recording method allows us to describe the potential muscular mechanism induced by the mucus in more detail, even if the biochemical mechanism underlying this response is still unknown. Namely, we clearly show that the entrance to the bursa copulatrix tract is closed off by waves of contractions of the copulatory canal rather than via a permanent closure of the tract. This is supported by the fact that these contractions were visible once every 10 minutes on average. With one exception within the Helicidae, our results indicate that the above-described reaction of the copulatory canal did not differ when the mucous extract of *C. aspersum* or one of the other species was applied. The non-specificity of this response is supported by two helicid species, *C. aspersum* and *T. pisana*, that share the recently discovered love-dart allohormone (LDA) that is the peptide contained in the mucous glands responsible for the contraction of the copulatory canal [44]. In addition, this effect of the copulatory canal may occur also in the Bradybaenidae family: when *Euhadra peliomphala* is injected with conspecific mucus, the bursa copulatrix tract of this species becomes inaccessible [16]. *Euhadra peliomphala* is a bradybaenid, just like *F. fruticum*, the only non-helicid in our study. This indicates that the function of dart mucus in closing off the tract leading to the bursa copulatrix may be a common strategy. Worth noting is that within the Helicidae, besides the two *Helix* species, the lowest percentage of copulatory canal response is found for *A. arbustorum*, a species for which dart shooting is facultative [45] and sperm storage does not increase in snails hit by the dart [46]. This suggests that the copulatory canal might not be strongly influenced by mucus in this species.

Overall, for both organs, we obtained a lower percentage of times the preparations responded to the mucous gland extract of *C. aspersum* compared to Koene and Chase [21]. This lower responsiveness might be due to the markers glued on the surface of the preparation affecting its activity. However, the markers did not prevent the preparation from moving, since waves of contractions were seen to spread through the entire length of the organs. The low activity might be due to an overestimation by Koene and Chase in quantifying the response, since our recorded videos showed that the diverticulum could be displaced by either the contracting copulatory canal or the atrium and vice versa (which is something that was hard to disentangle based on their method of recordings). In addition, to explain the difference in response measured for the diverticulum at the two time points, the acclimation period of the preparation in the saline solution immediately after dissection might not have been long enough for the diverticulum to adjust to the new solution. Consequently, this is likely to have influenced its stability (e.g. [47]). However, this does not affect the purpose of our study, since a relative response was used in our analyses and the responses of the preparations were all measured under the same experimental conditions.

It should be noted that in our tests, substances other than dart mucus were not tested. We decided not to include these here for several reasons. Firstly, Koene and Chase [21] used control extracts to test for general effects of muscle, connective tissue and mucous extracts; their mucus control being the pedal gland that releases mucus onto the snail’s foot during locomotion. That study demonstrated that the dart mucus was the most reliable substance to induce the contractions, but that the mucus of the pedal gland also evoked a similar effect in terms of number of contractions, even though the intensity was not measured. As they concluded, this indicates either that the active substance is a general constituent of mucus or that the two extracts cause a similar effect via different mechanisms. In this context, one can wonder about how likely it is that mucus from an entirely different source enters into the haemolymph via dart shooting. It is known that the love-dart is exclusively used to deliver mucous products into the mating partner (e.g. snails with excised mucous glands transfer a dry dart [20]). However, mucus present on the body wall might enter along with the mucous-covered dart albeit in insignificant quantities considering the small area of body wall that is wounded. Secondly, even if mucus from other sources also evokes a similar effect, this only indicates that the active component may also be used elsewhere in the body, in a different context than dart shooting. This is strongly suggested by the fact that the LDA precursor (see above) resembles buccalin precursors, which are known to be involved in modulation of muscle contractions in molluscs [44]. Moreover, the response induced on different parts of the body may also vary in strength. For example, in the grasshopper *Melanoplus sanguinipes* all male tissue extracts (e.g. brain, ventral nerve cord, haemolymph, accessory glands) induced contractions of the female oviduct with different degrees of response, the highest caused by the accessory gland complex [47]. Note that Koene and Chase [21] did not measure whether the intensity of contractions induced by pedal gland extracts was similar to the ones induced by dart mucus, hence a difference in intensity cannot be excluded. Thirdly, previous work on the focal species (*C. aspersum*) has reported repeatedly that mucous products delivered by the dart into the mating partner’s haemolymph are the ones responsible for the physiological changes that ultimately increase the paternity of the dart user, for example compared to controls with saline solution (e.g. [24, 48]). Given that most of the later studies did not include additional controls for nonspecific mucus effects, future work should aim to test such controls whenever relevant (e.g. when finding a new effect caused by dart mucus). Finally, with our study we now go beyond a single-species approach: we compare the response of *C. aspersum* to its own dart mucus with its response to those of other species in order to assess how specific the induced physiological changes are between different dart-bearing species. The proper way to test this is to contrast the known response (to mucus of the own/focal species) with the response to mucus from other species. Ideally, future research would include more recipient species in order to extend our understanding of the specificity of such male accessory gland products.

While the reactions of the diverticulum and copulatory canal were similar to the ones described by Koene and Chase [21], since contractions of both organs were easily observed simultaneously, a new response causing length reduction of the diverticulum is here presented for the first time. There is an advantage to produce such an effect, since there are two possibilities for sperm to escape the sperm digesting organ and reach the oviduct, either by elongating the spermatophore or by shortening the organ in which it is received [43]. A longer spermatophore’s tail, for example, protrudes into the vagina. Thus, sperm can safely exit the spermatophore through the tail [49]. Interestingly, the mucus of *Eobania vermiculata* causes the strongest shortening of *C. aspersum*’s diverticulum (almost 20 % of the total length). Among the species tested, *E. vermiculata* is the only one to possess a relatively long diverticulum with respect to its spermatophore-producing organs [50]. To overcome the difficulties that this long diverticulum imposes on the male, since the distance to the sperm storage organ increases, *E. vermiculata* could potentially benefit the most from the evolution of such a shortening effect. However, whether this advantage occurs within this species remains to be tested. Worth noting is that *F. fruticum*, belonging to a family without diverticulum, does not cause the shortening effect, strengthening our idea that substances targeting this organ may be exclusive to the Helicidae. The other species, for which this reaction was significant, might not benefit as much as *E. vermiculata* since they induce a much lower length reduction and because they have short diverticula and longer spermatophores. However, their response indicates that this effect is not species-specific.

Overall, the extent of the measured responses to each species’ mucus is not explained by phylogenetic relatedness, i.e. closely-related species do not resemble each other more in their response than less closely-related species, which contrasts with what would have been expected. Different degrees of manipulation between closely-related species could be expected if, under sexually antagonistic co-evolution, the female function of a species recently evolved a counter-adaptation (e.g. an increased threshold at which male substances are effective) and, as a consequence, the male function modified such manipulation to achieve its effect again (e.g. increased quantity of the substance).

Based on the current findings, we suggest that the responses to mucus carried by the love-dart in helicids snails are due to a mixture of bioactive substances, each targeting different organs and causing different effects. These substances induce responses that are similar between species, making the effect of mucus not species-specific. To strengthen such evidence, more effects need to be identified in other species as well as the peptides and proteins causing them. The lack of identification of mucous products (besides the LDA) prevent us from explaining whether the responses observed are due to similar or divergent substances possessed by the species tested. When the substances are different they can still have the same function [9, 27, 28]. This can, for example, be due to proteins with different amino acid sequences but similar tertiary structures [51].

Male manipulative substances can be diverse but their ultimate aim remains altering female behaviour or physiology. As a result, male substances in simultaneous hermaphrodites can also act similarly as in separate-sexed species. For example, land snails bearing love-darts cause a delay in remating of the partner [25] which is similar to what a *Drosophila* male does with females [11]. Comparable to the action of love-dart mucus reported here, studies on insects show that male products can also directly induce contractions of the female reproductive system (e.g. *Melanoplus sanguinipes* [47]; *Locusta migratoria* [52]). However, differences may occur in the evolution of such substances between different sexual systems. For instance, males can evolve manipulative substances that resemble female neuroendocrine molecules used for regulating processes of the reproductive system [53]. Males of separate-sexed species can evolve these female-like molecules through different mechanisms than simultaneous hermaphrodites, either via mutations or activation of silenced genes (reviewed in [54]). In contrast, the male function of simultaneous hermaphrodites has the great advantage to also possess female genes within the same individual. However, these genes still need to be expressed in male tissues before being exploited by the male function [55].

## Conclusions

Despite the lack of knowledge on the identity of mucous proteins carried on the dart of the different species, our study is the first to show that the love-dart transfers bioactive substances that seem functionally similar across most species’ mucus tested, based on the induced physiological changes measured here. While this indicates that these substances can act and evolve similarly in simultaneous hermaphrodites as in separate-sexed species, evolutionary differences between the two sexual systems need more attention and indeed more studies to confirm whether and when such differences are important.

Hence, the evolutionary framework that emerges from our findings provides important insight into the evolution of manipulative function of male products across species. Clearly, much more work is needed on different land snails species, and we see our study as a strong stimulus for many researchers to do such work on the species they investigate, thus filling the gaps in knowledge.

### Ethical Approval

No special ethical approvals were required to sample snails or to carry out our experiments.

### Availability of data

The datasets supporting the results of this article are available in the Dryad repository, http://dx.doi.org/10.5061/dryad.5rn52.

## Additional files

Additional file 1: Movie 1. Contractions of the diverticulum and copulatory canal of a *Cornu aspersum* preparation. This response occurs when mucous extract of *C. aspersum* is applied to the saline bath. Video sped up 16 times (original video length 10 min.). (MP4 4029 kb) Additional file 2: Movie 2. Shortening of *Cornu aspersum*’s diverticulum. This effect occurs immediately when the mucous extract of *Eobania vermiculata* is applied to the saline bath. Video sped up 8 times (original video length 50 sec.). (MP4 456 kb)
